# Advances in Organoid Culture Research

**DOI:** 10.1055/s-0042-1756662

**Published:** 2022-12-14

**Authors:** Zhiyuan Xie, Linghao Wang, Yan Zhang

**Affiliations:** 1 Med-X Research Institute, School of Biomedical Engineering, Shanghai Jiao Tong University, Shanghai, People's Republic of China; 2 State Key Laboratory of Oncogenes and Related Genes, Renji-Med X Stem Cell Research Center, Renji Hospital, School of Medicine, Shanghai Jiao Tong University, Shanghai, People's Republic of China

**Keywords:** organoid, material, microbiota, microbial metabolites

## Abstract

Organoids are powerful systems to facilitate the study of individuals' disorders and personalized treatments because they mimic the structural and functional characteristics of organs. However, the full potential of organoids in research has remained unrealized and the clinical applications have been limited. One of the reasons is organoids are most efficient grown in reconstituted extracellular matrix hydrogels from mouse-derived, whose poorly defined, batch-to-batch variability and immunogenicity. Another reason is that organoids lack host conditions. As a component of the tumor microenvironment, microbiota and metabolites can regulate the development and treatment in several human malignancies. Here, we introduce several engineering matrix materials and review recent advances in the coculture of organoids with microbiota and their metabolites. Finally, we discuss current trends and future possibilities to build more complex cocultures.

## Introduction

For decades, cancer cell lines have been the most common model used in vitro and have been successful in many areas, such as exploring the mechanism of cellular signaling pathways and researching potential drug targets. 1 Nevertheless, cell line models could not maintain the genetic phenotype and heterogeneity of the original tumor cells, let alone reproduce the morphology and function of the original tumor tissue. 2 Genetically engineered mouse models and patient-derived tumor xenograft (PDX) generated in mice are important advances in mimicking tumor niche, they can largely maintain the heterogeneity of the original tumor and the interaction between tumor and its surrounding stroma. 3 However, the establishment of the PDX model takes time, the genetic operation is difficult, and the cost of molding is expensive, which is not conducive to high-through drug screening. With a higher rate of success, shorter cycles, and better cost performance, organoids can make up for the defects of the above models and they more closely resemble the original tumor in vivo.

Organoids refer to tissue analogs with a certain spatial structure formed by the three-dimensional (3D) culture of adult stem cells or pluripotent stem cells in vitro. They can simulate the cell compositions, physiological functions, and genetic characteristics of tissues and organs in vivo to the greatest extent, as well as can be long-term stable subculture. 4 Organoid culture relies on basement membrane extract (BME), like Matrigel and collagen. However, these materials have poor physical and chemical properties, unclear composition, and uncontrollable stability between batches. In contrast, the physical and chemical properties of engineering matrix materials are relatively controllable, which is more conducive to organoid culture through adjustment and optimization.

Studies have shown that microbiota can reside within tumor cells and immune cells, impact the state of the tumor immune microenvironment, and even some microbial metabolites can also enter the bloodstream and modulate cancer cells and immune cells. 5 The introduction of microbiota or microbial metabolites into organoid culture can better reproduce the tumor microbe environment in vivo and study the occurrence and development of tumors more comprehensively. A deeper understanding of the microbiota-tumor-host relationship may also provide new prospects for cancer treatment and contribute to the development of new anticancer drugs.

In this review, we discuss the application of organoid in cancer research and highlight several engineering materials which have biomaterial properties for culturing organoids. Then, we illustrate the complex relationship between microbiota and cancer, and importantly, introduce how organoids can be used in the study of microbiota-tumor-host interactions.

### Organoids as a Model to Study Cancer

As early as 2009, a minigut organoid culture system was successfully established by adding R-spondin-1, epidermal growth factor, and Noggin into the Matrigel of mouse Lgr5 ^+^ intestinal stem cells, and intestinal organs with crypt-villus structures derived from adult stem cells were established based on this culture system. 6 The successful attempt and application of organoids in normal tissue make organoid culture technology develop rapidly in the field of tumor research. At present, various organoids have been successfully cultivated, including the intestine, liver, stomach, brain, kidney, heart, and skin. 7 8 9 10 11

The organoid model has a similar tissue arrangement and spatial structure to the donor organ and can simulate partial functions of the donor organ. It provides a research platform more similar to the environment in vivo for tumor research in vitro. The organoid model has become a new research tool for scientific problems that are difficult to be solved by traditional models, such as cancer genetics, cancer processes, and the activity of antitumor drugs. Organoid technology has broad application prospects in basic and clinical research, especially in disease pathology, cell biology, precision medicine, and drug toxicity. 12

Organoids allow the long-term expansion of normal and tumor tissues, and greatly preserve genetic diversity and morphological stability. There was heterogeneity in organoids cultured from tissues of different patient sources. 13 For the gastrointestinal and colorectal cancer (CRC) organoids, they show great similarity with the respective biopsies in morphology, the mutational spectrum, genes with an altered copy number, and expression patterns of common clinical diagnosis markers, 14 as well as they can be accurately reproduced in histopathology, hormone receptor status, and deoxyribonucleic acid (DNA) copy number variations. 15 This indicates that organoids can be used to study cell heterogeneity, construct biobanking, high-throughput drug screening, drug sensitivity test, etc., laying a foundation for the further development of personalized therapy and precision medicine.

Cancer is caused by the gradual accumulation of pathogenic gene mutations. Therefore, it is important to understand the mutational processes active in tissue homeostasis and tumorigenesis. Combined with CRISPR-Cas9 and orthotopic xenotransplantation in mice, cancer progression can be successfully regenerated in healthy tissue organoids, 16 17 the pathogenic genes and the mechanism of signaling pathways related to cancer progression can be explored by studying the progression of precancerous lesions. 18 19 Much effort has been exerted to model tumorigenesis and progression, to further elucidate the molecular mechanisms using tumor organoids.

However, compared with the physiological environment of normal tissues and organs, organoids still have limitations, such as a lack of connective tissue, vascular system, and immune cell. With the gradual development of precision medicine, it is believed that tumor organoids can be further developed to simulate tumors in vivo to a greater extent and become a powerful tool for personalized tumor therapy.

### Engineering Materials for Organoid Systems

Current organoid systems mostly rely on intrinsic or extrinsic biochemical signals (e.g., growth factors) and cell-cell interactions to control stem cell fate. 20 Organoid culture methods are complicated by the nearly exclusive dependence on animal-derived hydrogels, including Matrigel 21 and collagen, 22 as the 3D matrix. As a material derived from the secretion of Engelbreth–Holm–Swarm mouse sarcoma cells and enriched for biomimetic extracellular matrix (ECM) proteins, Matrigel is an ill-defined gelatinous basement membrane protein mixture composed of laminin, collagen IV, entactin, heparan sulfate, and numerous growth factors. 21 These matrices feature complex and variable compositions that are not conducive to controlled modifications, they can cause large batch-to-batch variations in cultured organoids. There are also potential risks of immunogen 23 and pathogen transfer, 24 making them unsuitable for organoid expansion in downstream clinical applications. In addition, Matrigel differed greatly from the ECM environment of normal tissues and organs, which could not provide a tissue-specific ECM environment for cells. 25 Also Matrigel is of high cost. Based on the above points, researchers aim at finding a replacement for Matrigel.

Natural polymer-based hydrogels are favored for their similarity to human ECM and their inherent biological activity. 26 Natural hydrogels are polysaccharides, proteins, and animal-derived mixtures. Polysaccharides are thought to be suitable for creating complex 3D scaffolds with multiple forms because of their rapid gelation properties and biodegradability. The typical polysaccharides are alginate, chitosan, dextran, cellulose, hyaluronic acid, agarose, pectin, gelatin, and heparin. Most natural hydrogels lack adequate mechanical strength, as well as a single type of hydrogel cannot meet all the requirements of organoids due to the complex and dynamic microenvironments in different tissues/organs in vivo. Therefore, many researchers believe that the combination of natural and synthetic hydrogels is a better choice for organoid culture.

Engineering synthetic hydrogels are combined with signaling proteins such as integrin-binding small molecules or bioactive peptides via chemical/enzymatic crosslinking for promoting the growth of stem cells and organoids. Synthetic hydrogels can provide chemically defined matrix, precisely adjust for matrix properties, and improve organoid culture efficiency and consistency. Synthetic hydrogels are easily produced with minimal batch variation by using traditional synthetic methods. 27 A variety of tumor organoids have been successfully cultured in engineering synthetic hydrogels. The common synthetic polymers for organoid culture are polyethylene glycol, nanocellulose, and other polymer matrices such as polyisocyanopeptide (PIC). 28 The development of materials capable of spatially and dynamically controlling organoid microenvironment and surrounding matrix makes it possible to regulate organoid maturation and function.

#### Nanocellulose-Based Hydrogel

Nanocellulose is a kind of excellent synthetic ECM suitable for organoid growth because of its hydrophilic, renewable, nontoxic, biodegradable, and biocompatible properties, as well as superior mechanical strength and modifiable surface. 29 Cellulose, as the precursor material of cellulose hydrogel, are biological macromolecules formed by glucose molecules connected by β-1,4-glycoside bonds. Cellulose molecules contain a large number of hydrogen bonds, which makes it a temperature-sensitive material. At low temperature, cellulose only form simple entanglement around hydrophobic groups without polymerization, but as the temperature increases, hydrogen bonds are destroyed, and their hydration gradually loses. The strong hydrophobic interaction between cellulose molecules leads to the formation of a 3D network. 30

Engineering nanocellulose hydrogel represents a performant and sustainable alternative for the organoid growth, and contributes to significantly reducing the costs in studies against diseases of global concern such as cancer. It has been reported that biologically active plant-based nanocellulose hydrogels functionalized with Arg-Gly-Asp (RGD) peptides and glycine supported the mouse intestinal organoids culture, 31 and can also be used for the breast cancer patient-derived organoids (PDOs) initiation and growth. 32

The proliferation and cell differentiation of organoids mainly depend on whether the hydrogel in which they are located can reconstruct the microenvironment of the base membrane. 33 Nanocellulose hydrogels are made of 99.9% water and 0.1% nanocellulose fibers by crosslinking with similar mechanical properties to the standard animal-based matrix, but do not provide any biological signal. Interestingly, they can be modified and functionalized due to the chemically reactive group in surface.

RGD peptides are the adhesive sites, as a fundamental element in matrices, which can induce cell attachment and differentiation. 34 Glycine, a nonpolar amino acid, is dissolved in the hydrogel to increase its osmolality, once the osmolality of the matrix is balanced by GLY, small intestinal crypts progress into cystic organoids. Based on this, organoids cultured in the RGD-GLY nanocellulose hydrogel sustain high viability, and even organoids can be formed from single cells, although the growth rate is considerably slower than those embedded in Matrigel. 31 When nanocellulose is blended with collagen (COL-NC) instead of RGD-GLY, intestinal crypts can form organoids with cystic structures, intact lumen, sphere-like morphology, and prominent epithelial budding. 35 PDOs grown in nanocellulose hydrogel synthesized by gelatin show similar proliferation, histopathologic features, gene expression, and drug responses to original tumors and PDOs formed in standard basement-membrane extract. 32

Besides the biological signal in hydrogels, the matrix stiffness also affects the formation and development of organoids, the mechanical properties of the hydrogels must resemble the stiffness of organs and tissues. 36 The stiffness of hydrogels is proportional to the type of solids and affected by the multiple cross-linkers and their concentration. 37 For example, the stiffness of COL-NC hydrogel is 90 Pa as same as Matrigel, while COL gels are much lower at 35 Pa 35 ; RGD-TPON hydrogels form a 3D network and are closer to that of Matrigel than RGD-PON hydrogels, which offer an optimum bioactive surface and appropriate stiffness for organoid attachment and growth. 37 Usually, nanocellulose hydrogels cross-linked with MgCl _2_ and CaCl _2_ , require four times more Mg ^2+^ than Ca ^2+^ ions to reach the stiffness of Matrigel. 37 The number of organoids in calcium cross-linked hydrogels sustains a massive decrease than the hydrogels cross-linked with Mg ^2+^ ions. It is important to select the right matrix materials and the type of cross-linkers agent to achieve the stiffness of a specific organ or tissue.

The modification of nanocellulose by physical, chemical, or biological methods can obtain suitable properties for organoid morphogenesis in vitro, which may fill the key gap of organoid technology lacking matrix glue.

#### Nonadhesive Alginate Hydrogels

Alginate is Food and Drug Administration-approved polysaccharide derived from algae and it is a favorable 3D culture material due to its biocompatibility and ease of manipulation with gelation and viscoelastic properties. Alginate hydrogels are structurally similar to the extracellular matrices of living tissue and can be prepared by a variety of chemical/enzymatic crosslinking methods. 38

Capeling et al 39 demonstrated that nonadhesive alginate hydrogel can support human intestinal organoid (HIO) growth in vitro. Hydrogels prepared by mixing alginate and gelatin can also exist in culture medium as suspended capsules for the cultivation of liver or intestinal organoids, and the mechanical and biological properties of this material are similar to those of tissue in vivo. 40 41 Alginate-grown HIOs are highly similar to Matrigel-grown HIOs in vitro, and the differentiation of epithelial cells is indistinguishable from HIOs grown in Matrigel. 39 Organoids cultured in suspended alginate hydrogels are closer to real tissues in vivo, as suspended capsules can effectively exchange nutrients with the surrounding medium to support the metabolism of multicellular clusters. 40 HIOs cultured in nonadherent alginate suspended capsules can form a serosal mesothelium that resembles the human fetal intestine. 41

Differentiation of stem cells is regulated by the stiffness of cell medium substrates. 42 Nonalginate hydrogel adjusts the mechanical strength of hydrogel by adjusting the proportion of compound composition and matching the mechanical strength of corresponding tissues in vivo, so as to be suitable for the culture of different types of organoids. 43 Nonadhesive alginate hydrogel can also form microbeads structure through microfluidic droplet devices, which can establish a high-throughput organoid model convent for drug screening. 44

Nonadhesive alginate hydrogel becomes a simple system and conducive to large-scale production due to a lack of adhesive or biochemical clues. It is a pity that the yield of alginate-grown organoids was significantly lower than in Matrigel. 39 At the same time, alginate cannot be degraded, which is not conducive to the recovery and passage of organoids. However, these shortcomings can be improved by adhesive/degradable modification. Alginate is also cost-effective. In conclusion, nonadhesive alginate hydrogel can be used to support 3D culture systems and advance regenerative medicine.

#### PIC-Based Hydrogels

PIC is a synthetic polymer and nonimmunogenic material, it can form thermosensitive hydrogels. It is a free-flowing liquid below 16°C, and the liquid becomes a viscous hydrogel within minutes when the temperature is above 16°C. 45

Like nanocellulose, PIC alone was not sufficient to support cell attachment or induce proliferation due to the lack of any bioactive component. When PIC hydrogels modify the adhesive RGD peptide, it can allow mammary gland organoids formation from breast fragments or purified single mammary epithelial cells 46 ; PIC hydrogels can also culture human liver organoids after modification with laminin-111. 47 The expansion and differentiation rate of organoids in PIC-based hydrogel is similar to that of Matrigel 48 ; while the potential for proliferation and differentiation could be maintained for many generations. 47 In addition, the proliferation rate depends on hardness, and lower hardness is the best condition for the proliferation of organoids. 47 Hydrogel hardness can also control colony formation efficiency. 46

PIC-based hydrogel is a chemically defined and synthetic hydrogel. The thermally reversible properties make it easier for cell recovery during organoid culture and make it beneficial for clinical applications such as cell therapy or tissue engineering. For example, PIC hydrogels have also been applied in vivo for subcutaneous cell transplantations and wound healing studies without any adverse effects. 49 50 These results highlight that cultured organoid combinations using well-defined synthetic hydrogels will pave the way for clinical applications in humans in the near future.

#### ECM Hydrogels Derived from Decellularized ECM

Decellularized tissue engineering is derived from the concept of tissue engineering. Its strategy is to use decellularization scaffolds of healthy organs as scaffold materials and then implant seed cells, such as stem cells, to induce the formation of organoids in a 3D culture system in vitro. 51 Decellularized matrix materials have attracted much attention in recent years because of their good biocompatibility, biodegradability, and tissue regeneration ability. 52 So far, reported in the article of the decellularized ECM (dECM) materials include skin, 53 muscle, 54 cartilage, 55 bone, 56 blood vessels, 57 lung, 58 liver, 59 kidney, 60 small intestine, 61 bladder, 62 trachea, 63 tissue dECM, etc. Some of them have been used in clinical treatment and have achieved good clinical efficacy.

Porcine gastric decellularized matrix (SEM) and intestinal decellularized matrix (IEM) hydrogel can culture gastric and intestinal (GI) organoids, respectively. 64 Hydrogels derived from healthy porcine liver ECM (PLECM) or human liver ECM (HLECM) can replace mouse tumor-derived BME for the culture and expansion of intrahepatic cholangiocyte organoids. 65 SEM and IEM hydrogels could effectively cultivate GI organoids with similar structure and functional characteristics as Matrigel organoids, even though some indexes were closer to natural tissues and organs, 64 let alone organoids in dECM hydrogels can be long-term culture, passage, and freezing storage. LECM hydrogels support the proliferation of bile duct cell-like organoids and maintain a bile duct cell-like phenotype, and no species-specific effects were observed between HLECM or PLECM hydrogels. 65

Although more and more dECM materials are being used in clinical therapy, dECM faces many challenges in its applications, including optimal decellularization schemes, degradation of materials, and regeneration regulation. The organoids cultured in dECM hydrogel had a low proliferation rate, and the intestinal villi/crypt structures of IEM are less than the organoids cultured in Matrigel. 64 But these limitations could be optimized by further improving protein retention other than collagen after decellularization. 66 So far, some cell fragments and nuclear residues still exist in the prepared dECM materials, even the dECM materials that have been commercialized and widely used in clinical practice. At the same time, nondegradation or incomplete degradation can hinder the growth of regenerative tissues and organs.

In summary, engineering matrices offer the possibility of customizing biochemical and mechanical properties according to organoid type and application that can support several different types of organoid cultures. This will become increasingly important once multiple organoid types need to be grown and maintained in a single cell culture system. However, these synthetic and highly defined hydrogels are still in their infancy. They can only be used in certain applications and are still not a complete replacement for widely used commercially available natural matrices.

### The Microbiota, Microbial Metabolites in Organoids

As understanding of the microbiota grows, we gradually understand the microbiota and microbial metabolites within mammalians. According to the classification of natural attributes, the microbiota within human has been identified in dozens of bacteria phyla, including *Bacteroidetes* , *Firmicutes* , *Proteobacteria* , *Actinobacteria* , *Verrucomicrobia* , *Fusobacteria* , and so on. 67 Ninety-eight percent of microbiota can be classified into the first four groups. Microbiota ferments dietary fiber and carbohydrates liberated from host mucins, and produces various fatty acids and amino acids through different metabolic pathways. 68 In addition, some gases and nitrogenous compounds are produced, particularly N-nitroso compounds. These metabolites accumulate in the bloodstream and have systemic effects on the host both protective and detrimental. 69 Accumulating evidence suggests that microbiota and microbial metabolites are involved in numerous biological processes, such as transformation process, tumor progression, and the response to anticancer therapies including immune checkpoint blockade. 70 71 72 73 74 75 76

To better dissect host-microbiota interactions, it is necessary to adopt models that mimic health and disease features as closely as possible. Traditional in vitro and in vivo experimental models are difficult to reproduce the host-microbiota relationship as represented by humans. Therefore, it is crucial to establish high-quality experimental models to understand the crosstalk of host, tumor, microbiota, and microbial metabolites. At present, many studies have reconstructed the complex tumor microenvironment in vivo by fusing immune cells, mesenchymal cells, and endothelial cells in organoids. If microbiota is fused, the complex physiological and pathological conditions of microbiota-tumor-host can be better simulated and the influence mechanisms of microbiota and their metabolites on tumor progression can be better studied. 77

#### Protumorigenic Microbiota and Microbial Metabolites

Microbiota can regulate some signaling pathways, affect the invasion and metastasis of tumor cells, and promote the occurrence and development of tumor. The Wnt/β-catenin and PI3K/AKT signaling pathway are involved in a wide range of biological processes, including cell migration, proliferation, differentiation, apoptosis, and immune response. 78 79 The alteration of these signaling pathways is a major cause of carcinogenesis. *Fusobacterium nucleatum* can activate the Wnt/β-catenin pathway through the upregulation of cyclin-dependent kinase 5 (Cdk5), and promote tumor cell proliferation and migration. 80 *Peptostreptococcus anaerobius* could drive CRC through increasing the gene expression and protein level of PI3K and AKT. 81

Microbial metabolites can also regulate tumor cell growth and affect the differentiation of immune cells. For example, formate can drive tumor progression and invasion by triggering AhR signaling, and regulating cancer stemness. 82 Butyrate may locally or systemically regulate the balance of anti-inflammatory and proinflammatory cytokines, and disrupt the ratio of regulatory T cells and Thelper17 cell subsets. 83 84 The high concentrations of secondary bile acids can be produced with gut microbiota enzyme and induce CRC. 85 86 And gut microbiota can use bile acids as a messenger to alter immune function and influence antitumor immunosurveillance. 87 Polyamines are mainly metabolized from arginine in host tissues, its synthesis also occurs in gut microbiota. 88 Polyamines are involved in a range of essential physiological functions, such as the maintenance of the structural integrity of membranes and nucleic acids, gene regulation, and translation. 89 90 Enterotoxigenic *Bacteroides fragilis* can upregulate polyamine production by host cells, 90 and affect the activity of various signaling pathways in tumor cells. 91

#### Antitumorigenic Microbiota and Microbial Metabolites

In addition to promoting tumor, most microbiota and their metabolites also inhibit tumor growth. A cocktail of *Lactobacillus spp* inhibits tumor cell growth via downregulating Wnt/β-catenin target genes. 92 Motility-associated killing factor A (MakA), a cytotoxin from *Vibrio cholera* , can induce cell apoptosis in several cancer cell lines, 93 like ileocecal cancer and colon carcinoma, and it can inhibit β-catenin-mediated tumor cell proliferation and reduce tumor burden via altering β-catenin integrity. *Leuconostoc mesenteroides* , isolated from dairy products, induced apoptosis and DNA fragmentation in a CRC cell line, as well as downregulated AKT in cells treated with bacterial conditioned media. 94 Some microbiota can induce tumor cells death by affecting the redox balance of tumor cells and upregulating genes related to oxidative stress response. 95 Indoles and their derivatives can regulate the differentiation of immune cells and increase the tolerance of the immune system. 96 97

Immune checkpoint block (ICB)-mediated antitumor responses mainly depend on cytotoxic T cells capable of recognizing and killing tumor cells. ICB has greatly improved the clinical efficacy of malignant tumor therapy and has great potential to be explored. 98 99 However, the immune response efficiency of ICB therapy is at a low level due to individual differences of patients. 100 It is necessary to find new ways to improve ICB response. More and more evidence indicate microbiota can improve the efficiency of ICB treatment efficiency. 101 102 For example, different types of *Bacteroides* may affect anticancer immunotherapy with CTLA-4 blockade. 103 *Bifidobacterium* and *Akkermansia muciniphila* can promote antitumor immunity and improve the therapeutic effect of anti-PD-L1 and anti-PD-1. 71 104 Microbial metabolites can also enhance the antitumor immune response and improve the immunotherapy response, such as inosine, polypeptide, and L-arginine. 105 106 107 108

#### Coculture of Microbiota and Organoids

At present, most research models of microbiota are based on germ-free mice, 109 which have two defects: first, the cells and microbiota of this model are all from mice, which are different from human species; Second, due to the extremely complex microbiota and its effects, mouse models cannot be finely controlled in the experimental process. Therefore, it is difficult to obtain more detailed and in-depth results of mechanism properties. Organoids can be used to establish in vitro research models of complex microbial communities in direct contact with the mucus layer of human intestinal cells, complementing the significant deficiencies of mouse models in experimental control, scalability, and reproducibility of interactions between the human gut and host-specific symbiotic microbiota. It is extremely valuable for further study of the interaction between host and microbiota.

The coculture technology of organoids with microbiota has been used widely and made substantial progress in disease research. Engevik et al 110 found colony-stimulating factor (CSF) induce interleukin-10 expression in dendritic cells and regulate the host immune system by coculture of *Lactobacillus reuteri* , organoids, and dendritic cells. A genotoxic polyacetyl-1-peptide (colibactin) in PPKS ^+^ *Escherichia coli* that causes DNA double-strand cross-linking and damage which is associated with CRC, 111 this study provides important evidence for further treatment and research of CRC through studying the direct effect and causal relationship between gut microbiota genotoxic substances and oncogene mutations. To investigate how the tumor-resident microbiota influence the tumor cell activity, researchers established a coculture system of organoids with microbiota, 112 organoids imaging results showed that microbiota help tumor cells survive in the circulatory system by regulating the cytoskeleton, thereby promoting tumor metastasis.

Systematic protocols have been developed to guide the coculture of intestinal organoids with microbiota. 113 Organoids can form lumens that represent the intestinal lumen, microbiota was injected into lumen by microinjection and represent faithfully the microbiota–epithelium orientation. This method was successfully used to inoculate human gastric organs with *Helicobacter pylori* . 114 Microinjection is a technical skill, it will be failed if the organoids are too small, too differentiated, too dense, or the microbes are too overgrown, and most of the intestinal organoids have to be as similar in size as possible. This operation also does not allow for high-throughput scaling, one of the advantages is that it is more in line with the actual physiological state of the body.

Importantly, organoids coculture with single microbiota does not take into account the overall complexity of the gut microbiota ecosystem, such as complex interactions with immune compartments. The intestinal organoids and organs-on-chips 115 approach has made significant progress and can achieve long-term coculture with a variety of microbiota species. Jalili-Firoozinezhad et al 116 developed a two-channel microfluidic organoid chip device, Intestine Chip System, the system has the capability of maintaining a complex microbiota community in coculture with human intestinal epithelial cells, and to study host-microbiota interactions through direct contact with human intestinal epithelial cells via an in vitro covering simulated mucous layer. Compared with aerobic culture conditions, microfluidic chip-controlled chamber of oxygen gradient increased the intestinal barrier function and provides maintenance of microbiota diversity related to physiological levels, including more than 200 from 11 different genera classification of obligate anaerobic bacteria. Microfluidic organoid chip can be used as a tool to develop microbiota-related therapies, probiotics, and nutrients.

## Conclusion

As 3D micro-organs cultivated in vitro, organoids have the potential to construct human organ disease models. They are recognized as important tools in biological research and can make up for the shortcomings of cell lines and animal models in clinical application. Organoid research is still in its infancy and there are many problems to be solved. For example, animal-derived matrix materials used to support organoid growth are not conducive to organoid expansion in downstream clinical applications due to their complex and variable components, differences between batches, and risks of immunogen and pathogen transfer. Engineering hydrogels can be synthesized using traditional methods to provide chemically well-defined matrices that can be precisely tuned to improve organoid culture efficiency and consistency. Compared with Matrigel, the engineering materials currently developed have a slightly lower efficiency in organoid culture, but they still have broad development and application prospects.

Microbiota and microbial metabolites play a key role in human health and disease. Different types of microbiota and microbial metabolites play different roles in the occurrence and development of tumors, immunology, and cancer therapy. But it is still having a lot to learn for the internal mechanism and how to adjust the microbiota to enhance response to cancer immunotherapy. Traditional experimental models cannot well simulate the relationship between host and microbiota, so it is difficult to obtain more detailed and in-depth results of mechanism properties. To better analyze the host-microbiota interaction, microbiota and organoid technology can be combined to reconstruct the complex tumor microenvironment in vivo, providing new details for studying the occurrence and development mechanism of various diseases including cancers. At present, the application of microbiota and organoid coculture technology has played an important role in the study of some diseases, opening up new possibilities for understanding and treating diseases. However, this coculture model is still in its infancy and there is much more space for improvement in the future.
